# Bacterial Biohybrid Microswimmers

**DOI:** 10.3389/frobt.2018.00097

**Published:** 2018-08-29

**Authors:** Julio Bastos-Arrieta, Ainhoa Revilla-Guarinos, William E. Uspal, Juliane Simmchen

**Affiliations:** ^1^Physikalische Chemie, Technische Universität Dresden, Dresden, Germany; ^2^Department of General Microbiology, Institute of Microbiology, Technische Universität Dresden, Dresden, Germany; ^3^Department of Theory of Inhomogeneous Condensed Matter, Max-Planck-Institut für Intelligente Systeme, Stuttgart, Germany; ^4^IV. Institut für Theoretische Physik, Universität Stuttgart, Stuttgart, Germany

**Keywords:** bacteriabots, microswimmers, bacterial surface, bacterial motility, surface charge, binding

## Abstract

Over millions of years, Nature has optimized the motion of biological systems at the micro and nanoscales. Motor proteins to motile single cells have managed to overcome Brownian motion and solve several challenges that arise at low Reynolds numbers. In this review, we will briefly describe naturally motile systems and their strategies to move, starting with a general introduction that surveys a broad range of developments, followed by an overview about the physical laws and parameters that govern and limit motion at the microscale. We characterize some of the classes of biological microswimmers that have arisen in the course of evolution, as well as the hybrid structures that have been constructed based on these, ranging from Montemagno's ATPase motor to the SpermBot. Thereafter, we maintain our focus on bacteria and their biohybrids. We introduce the inherent properties of bacteria as a natural microswimmer and explain the different principles bacteria use for their motion. We then elucidate different strategies that have been employed for the coupling of a variety of artificial microobjects to the bacterial surface, and evaluate the different effects the coupled objects have on the motion of the “biohybrid.” Concluding, we give a short overview and a realistic evaluation of proposed applications in the field.

## Introduction

Artificial micro- and nano-swimmers are small scale devices that convert energy into movement (Ozin et al., 2005; Wang and Pumera, 2015). Since the first demonstration of their performance in 2002, the field has developed rapidly in terms of new preparation methodologies, propulsion strategies, motion control, and envisioned functionality (Ismagilov et al., 2002; Katuri et al., 2017). This very active field of research holds promises for many different applications, with drug delivery, environmental remediation, and sensing being some of the most remarkable. While the initial focus of the field was largely on purely artificial systems, lately, an increasing number of “biohybrids” have appeared in the literature. Combining artificial and biological components is a promising strategy to obtain new, well-controlled microswimmer functionalities, since essential functions of living organisms are intrinsically related to the capability to move (Vale and Milligan, 2000). Living beings of all scales move in response to environmental stimuli (e.g., temperature or pH), to look for food sources, to reproduce, or to escape from predators. One of the most well-known living microsystems are swimming bacteria, but directed motion occurs even at the molecular scale, where enzymes and proteins undergo conformational changes in order to carry out biological tasks (Vogel, 2005). Since Nature has been optimizing strategies, principles, and methods of locomotion over millions of years, it seems only logical that scientists should seek to imitate biological principles in synthetic systems, or—even further—directly couple biological and artificial components to obtain motile biohybrids.

This review starts with some scenes from the historical discovery and formulation of the physical laws that determine the modes of motility on the small scale and have to be obeyed by both artificial and natural swimmers. In the subsequent sections, we present the main motile entities found in nature, and then specify more details about bacteria, as they are the main focus of this review. Building on this microbiological foundation, we go more into detail about the coupling strategies that have been used to create bacterial hybrid microswimmers. Later, we discuss the effects of the shape of the artificial components of the biohybrid on speed and other properties of motion. Finally, we conclude this manuscript by introducing some of the first applications that have been presented in the literature.

### Physical principles related to motion at the microscale

In 1828, the British biologist Robert Brown discovered the incessant jiggling motion of pollen in water and described his finding in his article “A Brief Account of Microscopical Observations…” (Brown, 1828), leading to extended scientific discussion about the origin of this motion. This enigma was resolved only in 1905, when Albert Einstein published his celebrated essay “Über die von der molekularkinetischen Theorie der Wärme geforderte Bewegung von in ruhenden Flüssigkeiten suspendierten Teilchen” (Einstein, 1905). Einstein not only deduced the diffusion of suspended particles in quiescent liquids, but also suggested these findings could be used to determine particle size—in a sense, he was the world's first microrheologist.

Ever since Newton established his equations of motion, the mystery of motion on the microscale has emerged frequently in scientific history, as famously demonstrated by a couple of articles that should be discussed briefly. First, an essential concept, popularized by Osborne Reynolds, is that the relative importance of inertia and viscosity for the motion of a fluid depends on certain details of the system under consideration. The Reynolds number *Re*, named in his honor, quantifies this comparison as a dimensionless ratio of characteristic inertial and viscous forces:

(1) $$\begin{array}{r} Re=\frac{\rho Vl}{\eta } \end{array}$$

Here, ρ represents the density of the fluid; *V* is a characteristic velocity of the system (for instance, the velocity of a swimming particle); *l* is a characteristic length scale (e.g., the swimmer size); and η is the viscosity of the fluid. Taking the suspending fluid to be water, and using experimentally observed values for *V*, one can determine that inertia is important for macroscopic swimmers like fish (Re = 100), while viscosity dominates the motion of microscale swimmers like bacteria (Re = 10^−4^).

The overwhelming importance of viscosity for swimming at the micrometer scale has some profound implications for swimming strategy, as discussed most memorably by Purcell. He invited the reader into the world of microorganisms and theoretically studied the conditions of their motion (Purcell, 1977).

In the first place, propulsion strategies of large scale swimmers often involve imparting momentum to the surrounding fluid in periodic, discrete events (e.g., by vortex shedding), and coasting between these events through inertia. This cannot be effective for microscale swimmers like bacteria: due to the large viscous damping, the inertial coasting time of a micron-sized object is on the order of 1 μs. Using the typical speed of a microorganism, one obtains that the coasting distance is 0.1 Å. Purcell concluded that only forces that are exerted in the present moment on a microscale body contribute to its propulsion, so a constant energy conversion method is essential. Microorganisms have optimized their metabolism for continuous energy production, while purely artificial microswimmers must obtain energy from the environment, since their on-board-storage-capacity is very limited. As a further consequence of the continuous dissipation of energy, biological and artificial microswimmers do not obey the laws of equilibrium statistical physics, and need to be described by non-equilibrium dynamics. Mathematically, Purcell explored the implications of low Reynolds number by taking the Navier Stokes equation and eliminating the inertial terms:

(2) $$\begin{array}{r} -\nabla \rho +\eta \nabla^{2}v=0 \end{array}$$

As he noted, the resulting equation—the Stokes equation—contains no explicit time dependence. This has some important consequences for how a suspended body (e.g., a bacterium) can swim through periodic mechanical motions or deformations (e.g., of a flagellum). First, the *rate* of motion is practically irrelevant for the motion of the microswimmer and of the surrounding fluid: changing the rate of motion will change the *scale* of the velocities of the fluid and of the microswimmer, but it will not change the pattern of fluid flow. Secondly, reversing the direction of mechanical motion will simply reverse all velocities in the system. These properties of the Stokes equation severely restrict the range of feasible swimming strategies. As a concrete illustration, consider a “scallop” that consists of two rigid pieces connected by a hinge. Can the scallop swim by periodically opening and closing the hinge? No: regardless of how the cycle of opening and closing depends on time, the scallop will always return to its starting point at the end of the cycle. Here originated the famous quote: “Fast or slow, it exactly retraces its trajectory and is back where it started.” In light of this “scallop theorem,” Purcell developed approaches concerning how artificial motion at the micro scale can be generated. This paper inspires scientific discussions until today; for example, a recent work of the Fischer group experimentally confirmed that the scallop principle is only valid for Newtonian fluids (Qiu et al., 2014).

### Biological motile systems

**Figure 1:**
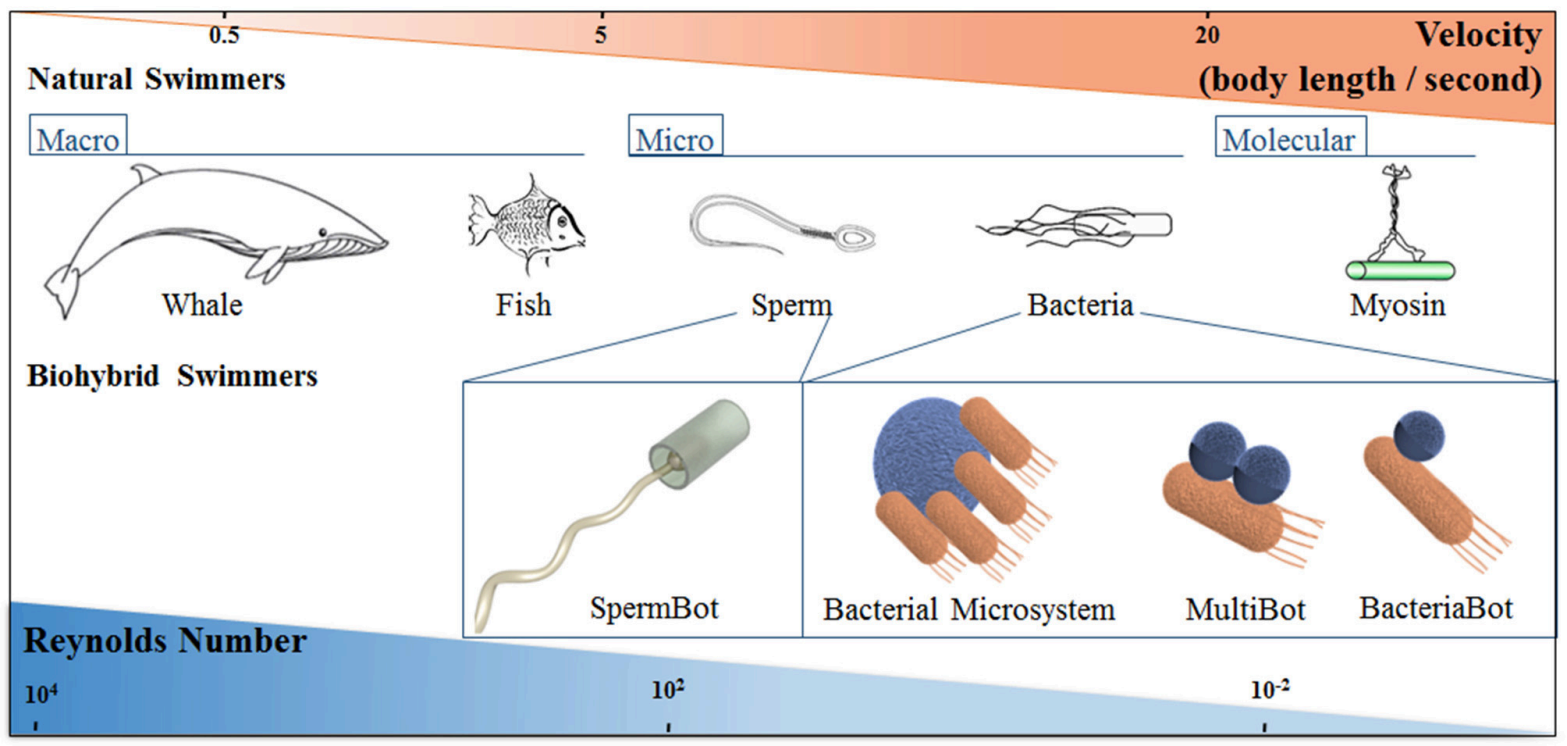
Schematic representation of natural and biohybrid swimmers on different length scales, showing how the characteristic swimmer velocity and Reynolds number changes with length scale. The classification of Bacterial biohybrid microswimmers according to the bacteria: object ratio is indicated; see the main text for details.

Nature has developed motile systems over time and length scales spanning several orders of magnitude, which have evolved anatomically and physiologically to attain optimal strategies for self-propulsion and overcome the implications of high viscosity forces and Brownian motion, as shown in Figure 1 (Lauga and Powers, 2009).

Some of the smallest known motile systems are motor proteins, i.e., proteins and protein complexes present in cells that carry out a variety of physiological functions by transducing chemical energy into mechanical energy. These motor proteins are classified as myosins, kinesins, or dyneins. Myosin motors are responsible for muscle contractions and the transport of cargo using actin filaments as tracks. Dyneins and kinesin motors, on the other hand, use microtubules to transport vesicles across the cell (Vogel, 2005; Patra et al., 2013). The mechanism these protein motors use to convert chemical energy into movement depends on ATP hydrolysis, which leads to a conformation modification in the globular motor domain, leading to directed motion (Feringa, 2001; Schliwa and Woehlke, 2003).

Specifically, for microorganisms that live in aqueous environments, locomotion refers to swimming, and hence our world is full of different classes of swimming microorganisms, such as bacteria, spermatozoa, protozoa, and algae. Some biological microswimmers are listed here:

Bacteria: move due to rotation of hair-like filaments (flagella) anchored to a protein motor complex on the bacteria cell wall. More detailed information about bacteria will be discussed in section Bacterial Biohybrid Microswimmers, as the main focus of this review (Berg, 2003).
Spermatozoa: if the male reproductive cell is (uni) flagellated and motile, it is referred to as a spermatozoon, a phenotype which is dominant in animals. Many uniflagellar spermatozoa are propelled by a snake-like wiggling of their flagella. This flagellar beat is a wave that propagates from the head of the spermatozoon to the tip of the flagellum (Friedrich et al., 2010).
Paramecia (unicellular protozoa): contain many hair-like extrusions all over the body (cilia). The ciliar beat has two distinct phases: the *Power stroke*, in which the cilium is stretched out straight and moves rather fast in one direction; and the *Recovery stroke*, in which each cilium bends and twists a little sideways while it slowly retracts (Lindemann and Lesich, 2010; Lindemann, 2014).
Motile algae: belonging to the filo Euglenophyta, these flagellated cells swim in a coordinated fashion, synchronizing one or several flagellar structures to achieve self-propulsion. Since most of these organisms undergo photosynthesis, many of them have eye-spots near the anterior pole to enable the algae colony to swim toward light (Drescher et al., 2010).

### Hybrid bioswimmers

Hybrid biomicroswimmers can be defined as microswimmers that consist of both biological and artificial constituents, for instance, one or several living microorganisms attached to one or various synthetic parts (Schwarz et al., 2017).

The pioneers of this field, far ahead of their time, were Montemagno and Bachand with a work regarding specific attachment strategies of biological molecules to nanofabricated substrates enabling the preparation of hybrid inorganic/organic nano-electro-mechanical systems, so called NEMS (Montemagno and Bachand, 1999). They described the production of large amounts of F_1_-ATPase from *Bacillus* PS3 for the preparation of F_1_-ATPase biomolecular motors immobilized on a nanoarray pattern of Au, Cu or Ni produced by electron beam lithography. These proteins were attached to 1 μm microspheres tagged with a synthetic peptide. Consequently, they accomplished the preparation of a platform with chemically active sites and the development of biohybrid devices capable of converting energy of biomolecular motors into useful work.

Recent publications of biohybrid microswimmers include the use of sperm cells, contractive muscle cells, and bacteria as biological components, as they can efficiently convert chemical energy into movement, and additionally are capable of performing complicated motion depending on environmental conditions. In this sense, biohybrid microswimmer systems can be described as the combination of different functional components: cargo and carrier. The cargo is an element of interest to be moved (and possibly released) in a customized way. The carrier is the component responsible for the movement of the biohybrid, transporting the desired cargo, which is linked to its surface. The great majority of these systems rely on biological motile propulsion for the transportation of synthetic cargo for targeted drug delivery (Schwarz et al., 2017). However, some examples of the opposite case—artificial microswimmers with biological cargo systems—will not be explained in detail here since they are out of the focus of this review (Wu et al., 2014; Wang and Pumera, 2015).

The carrier for a biohybrid microswimmer must be capable of generating a thrust that can balance the viscous drag on the whole assembly, and therefore produce directed motion. The role of a biological carrier can be accomplished by two types of cells: motile entities (e.g., spermatozoa and bacteria) and muscle cells (Carlsen and Sitti, 2014; Wang and Pumera, 2018).

Sperm cells have been shown to be a suitable biological carrier in biohybrid devices due to their motility and directional guidance by chemotaxis, thermotaxis, and rheotaxis. Magdanz et al. created the first reported biohybrid spermbot by trapping sperm cells in magnetic microtubes (Figure 1), leading to a system with the ability to be remotely guided using an external magnetic field. This approach has significant potential for the design of novel assisted reproductive protocols (Magdanz et al., 2013, 2017). Further studies regarding Spermbots used polymer microhelices with a soft magnetic NiTi bilayer coating for the capture, transport, and release of single immotile live sperm cells (Medina-Sánchez et al., 2016).

Biohybrid microswimmers have incorporated muscle (cardiac, smooth, and skeletical) cells or myocytes, due to their capability of spontaneous contraction with no external driving stimulus. The power generated from the contractions is proportional to the cross-sectional area of the muscle cell-tissue assembly. Moreover, the contraction state can be prolonged when suitable light or electric pulses are applied, leading to increased tuneability (Asano et al., 2012; Carlsen and Sitti, 2014). In 2014, Williams and co-workers presented the preparation of a biohybrid microswimmer based on cardiomyocytes cultured on a synthetic polydimethylsiloxane filament with a short rigid head that propels at 5–10 μm s^−1^ (Williams et al., 2014).

As previously mentioned, this review is focused on different aspects of bacteria as the biological component of a biohybrid microswimmer, referring to the case in which bacteria act as the biological carrier of biohybrid microswimmers (Figure 1). Thus, the bacterial cells are responsible for motility while attached to synthetic inorganic functional components. In order to have a clear terminology we define: a BacteriaBot has a 1:1 ratio of bacteria:cargo, a MultiBot refers to one bacteria attached to multiple cargoes, while a BacterialMicrosystem refers to several bacteria:one cargo. The attachment of multiple cargoes to one bacteria has only been reported in a few cases, but can regardless be considered a MultiBot. A case that should be only marginally discussed here are collective/turbulence driven systems. Here, the ratio is not definable, but one (or more) cargoes are moved by the flow created by many bacteria. Thereby we partially adapt the nomenclature indicated by Martel and colleagues (Martel, 2012).

## Bacterial biohybrid microswimmers

Bacteria are found in all terrestrial and aquatic environments that support life. They are prokaryotic microorganisms with different sizes (ranging from 0.5 to 5 μm in length) and different morphologies: spherical or ovoid cell shape, called *coccus* (plural *cocci*), cylindrical shape, called *rod* and spiral cell shape (*spirilla*), amongst others (Young, 2006). Bacteria can exist as single cells, but also associated (for instance in chains) or in multicellular structures like aggregates and hyphae (Madigan et al., 2014). Many single bacteria cells can move using their own power. Altogether, bacteria offer a wide catalog of properties that could be—and are starting to be—exploited for their use in hybrid microswimmers.

Bacterial biohybrid microswimmers, composed of at least one living bacteria and one inanimate object, are the focus of a vibrant and quickly growing research field. So far, prototypes for a number of very different options have been implemented: the motile force can come from a single or many bacteria (Di Leonardo et al., 2010; Barroso et al., 2015) or the bacteria can be moved by a motorized object (Campuzano et al., 2012). Different designs varied the bacteria—object ratio: from one bacterium—one object (Stanton et al., 2016; Park et al., 2017), via one bacterium—many objects (Akin et al., 2007; Luo et al., 2016), to many bacteria—one object (Darnton et al., 2004; Kim et al., 2012). From the microbiological point of view, two main characteristics of bacteria must be considered when discussing bacterial biohybrid microswimmers: (i) their surface properties, which control cargo attachment, and (ii) their motile abilities, i.e., whether the motive force of the bacteria is sufficient to move the cargo from a point A to B.

### Bacterial surface

Bacteria can be roughly divided into two fundamentally different groups—called Gram-positive and Gram-negative bacteria—distinguished by the architecture of their cell envelope, in both cases a complex multi-layered structure that protects the cell from its environment. In Gram-positive bacteria, the cytoplasmic membrane is only surrounded by a thick cell wall of peptidoglycan. In contrast, the envelope of Gram-negative bacteria is more complex and consists (from inside to outside) of the cytoplasmic membrane, a thin layer of peptidoglycan, and an additional outer membrane, also called the lipopolysaccharide layer. Other bacterial cell surface structures include capsules and slime layers, which are secreted slimy or sticky materials made up of polysaccharides or proteins that cover the cells and are in direct contact with the environment; they have different functions, including attachment to solid surfaces. Additionally, protein appendages can be present on the surface: fimbriae and pili can have different lengths and diameters and their functions include adhesion and twitching motility (Madigan et al., 2014; Dufrêne, 2015).

The cytoplasmic membrane is a thin (6–8 nm) and fluid phospholipid bilayer, which also contains numerous membrane-embedded and membrane-associated proteins. The membrane surrounds the cell separating the cytoplasm from the cell's environment and it is a selective permeability barrier for substances that enter and exit the cell (Madigan et al., 2014). Protons (H^+^) and hydroxyl ions (OH^−^) cannot diffuse across this membrane, allowing a charge separation that enables the proton motive force which is used to generate energy for different cell functions, including motility (Manson et al., 1977). Surrounding the cytoplasmic membrane is the cell wall that protects the cell against osmotic lysis, provides shape and rigidity, and is a protective layer against toxic substances. The cell wall is composed of peptidoglycan, a polysaccharide composed of alternating residues of β-1-4-linked N-acetylmuramic acid (NAM) and N-acetylglucosamine (NAG), cross-linked by short peptides (Vollmer et al., 2008). The glycan strands run circumferentially around the cell and are cross-linked by the peptides running approximately parallel to the long axis of the cell (Beeby et al., 2013); both glycan strands and associated stem peptides are usually modified (Vollmer, 2008).

In Gram-positive cell walls, the number of peptidoglycan layers is variable (20–80 nm thick). The cell wall may contain polysaccharides and proteins anchored through different mechanisms, and teichoic acids (TAs), covalently attached to peptidoglycan (wall teichoic acids, WTAs) or anchored to membrane lipids (lipoteichoic acids, LTAs), threading through the peptidoglycan layers. TAs are long anionic polymers of repeating units of glycerol phosphate, glucosyl phosphate, or ribitol phosphate, and they are partially responsible for the overall negative surface charge of the Gram-positive cell envelope (Neuhaus and Baddiley, 2003). In the field of biohybrid microswimmers, the negative surface charge of Gram-positive *Bacillus subtilis* cells was used to electrostatically attach zeolite L crystals chemically modified with amino groups, leading to positively charged surfaces (Barroso et al., 2015). Other Gram-positive bacteria that have been used to develop bacterial hybrid swimmers are *Listeria monocytogenes* (Akin et al., 2007), *Bifidobacterium breve* and *Clostridium difficile* (Luo et al., 2016).

In Gram-negative bacteria the peptidoglycan wall is thinner (between 1.5-15 nm) and there is an outer membrane anchored to the peptidoglycan through lipoproteins (Braun, 1975; Vollmer et al., 2008). The inner half of the outer membrane contains lipoproteins and phospholipids; in the outer half of the outer membrane, the lipopolysaccharide, composed of lipid A, the core polysaccharide and the O-antigen polysaccharide, replaces much of the phospholipid (Silhavy et al., 2010). The main function of the outer membrane is structural, and its presence on the cell envelope of Gram-negative bacteria creates another cell compartment, the periplasmic space. It is located between the outer surface of the cytoplasmic membrane and the inner surface of the outer membrane and contains many proteins involved in different cellular processes like nutrient transport, chemotaxis, and envelope biogenesis (Ehrmann et al., 2007). Unlike the few Gram-positive bacteria listed above, many Gram-negative bacteria have been widely used in the development of hybrid microswimmers. This includes *Vibrio alginolyticus* (Sowa et al., 2003; Nogawa et al., 2010; Kojima et al., 2013; Zhang et al., 2013), *Serratia marcescens* (Darnton et al., 2004; Behkam and Sitti, 2006; Steager et al., 2007, 2011; Park et al., 2010; Traoré et al., 2011; Carlsen et al., 2014; Kim and Kim, 2016), *Magnetococcus marinus* MC-1 (Felfoul et al., 2011, 2016), *Salmonella typhimurium* (Cho et al., 2012; Park et al., 2013a; Li et al., 2015) and *Escherichia coli* (Di Leonardo et al., 2010; Singh and Sitti, 2016; Stanton et al., 2016; Suh et al., 2016; Park et al., 2017). The different strategies applied to couple a cargo to the bacterial surfaces will be further discussed in section Binding Strategies for the Preparation of BacteriaBots.

### Bacterial locomotion

**Figure 2:**
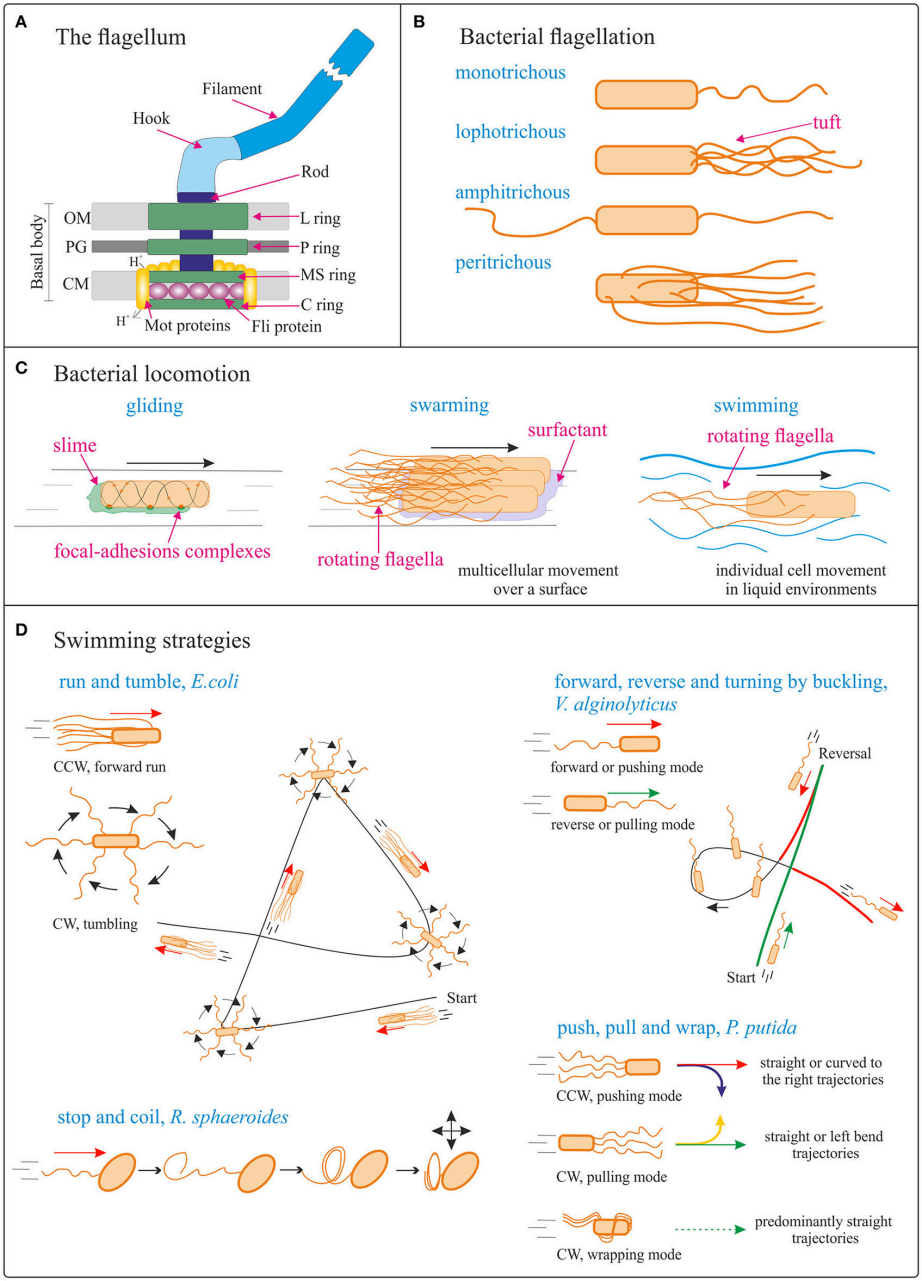
Bacterial locomotion. **(A)** Schematic (not to scale) structure of the flagellum in Gram-negative bacteria. OM, outer membrane; PG, peptidoglycan cell wall; CM, cytoplasmic membrane. Adapted from Madigan et al. (2014). **(B)** Different types of bacterial flagellation. **(C)** Different bacterial locomotion strategies: gliding (focal adhesion model) (adapted from Islam and Mignot, 2015), swarming (adapted from Kearns, 2010), and swimming. The direction of bacteria movement is indicated by black arrows. **(D)** Schematics (not to scale) of different swimming strategies. Run and tumble from *Escherichia coli*; forward, reverse, and turning by buckling of *Vibrio alginolyticus* (adapted from Son et al., 2013); stop and coil from *Rhodobacter sphaeroides* (adapted from Armitage and Macnab, 1987; Armitage et al., 1999); push, pull, and wrap from *Pseudomonas putida* (adapted from Hintsche et al., 2017). The direction of bacteria movement is indicated by the arrows. See main body text for details.

Most rod-shaped bacteria can move using their own power, which allows colonization of new environments and discovery of new resources for survival. Bacterial movement depends not only on the characteristics of the medium, but also on the use of different appendages to propel. *Swarming* and *swimming* movements are both powered by rotating flagella (Figure 2C) (Berg and Anderson, 1973; Turner et al., 2010). Whereas swarming is a multicellular 2D movement over a surface and requires the presence of surfactant substances, swimming is movement of individual cells in liquid environments (Henrichsen, 1972). These two types of bacterial motility are the most relevant for the development of bacterial biohybrid microswimmers and will be described in more detail below. Other types of movement occurring on solid surfaces include twitching, gliding and sliding, which are all independent of flagella. *Twitching* motility depends on the extension, attachment to a surface, and retraction of type IV pili which provide the energy required to push the cell forward (Mattick, 2002). *Gliding* motility uses a highly diverse set of different motor complexes, including e.g., the focal-adhesion complexes of *Myxococcus* (Figure 2C) (Islam and Mignot, 2015; Nan and Zusman, 2016). The gliding bacterium *Mycoplasma mobile* (lacking a peptidoglycan layer; Razin et al., 1998) was used to transport 0.5 μm streptavidin conjugated beads (Hiratsuka et al., 2005) and to power in a predefined direction a streptavidin coated microrotary motor composed of a 20 μm-diameter silicon dioxide rotor driven on a silicon track (Hiratsuka et al., 2006). Unlike twitching and gliding motilities, which are active movements where the motive force is generated by the individual cell, *sliding* is a passive movement. It relies on the motive force generated by the cell community due to the expansive forces caused by cell growth within the colony in the presence of surfactants, which reduce the friction between the cells and the surface (Hölscher and Kovács, 2017).

#### Flagella: conversion of electrochemical energy into mechanical work

The flagellum (plural, *flagella*; a group of flagella is called a *tuft*) is a helical, thin and long appendage attached to the cell surface by one of its ends, performing a rotational motion to push or pull the cell (Berg and Anderson, 1973). Different types of cell flagellation are found depending on the number and arrangement of the flagella on the cell surface, e.g., only at the cell poles or spread over the cell surface (Figure 2B; Leifson, 1960). In polar flagellation, the flagella are present at one or both ends of the cell: if a single flagellum is attached at one pole, the cell is called *monotrichous*; if a tuft of flagella is located at one pole, the cells is *lophotrichous*; when flagella are present at both ends, the cell is *amphitrichous*. In *peritrichous* flagellation, the flagella are distributed in different locations around the cell surface. Nevertheless, variations within this classification can be found, like lateral and subpolar—instead of polar—monotrichous and lophotrichous flagellation (Haya et al., 2011).

The flagellum is a complex protein-nanomachine that uses an electrochemical gradient (of H^+^ or Na^+^ ions) to perform mechanical work (Figure 2A) (Manson et al., 1977; Hirota et al., 1981; Elston and Oster, 1997). For detailed description of its structure and working mechanism interested readers are referred to specific reviews (Berg, 2003; Erhardt et al., 2010; Evans et al., 2014; Minamino and Imada, 2015). In a nutshell, the flagellum is composed of three parts: the basal body, the hook, and the filament (Figure 2A). The basal body is a reversible motor that spans the bacterial cell envelope. It is composed of the central rod and several rings: in Gram-negative bacteria, these are the outer L- (“lipopolysaccharide”) and P- (“peptidoglycan”) rings, and the inner MS- (“membrane/supramembrane”) and C- (“cytoplasmic”) rings. In Gram-positive bacteria only the inner rings are present (Chen et al., 2011). The Mot proteins surround the inner rings in the cytoplasmic membrane; ion translocation through the Mot proteins provide the energy for flagella rotation (Manson et al., 1977). The Fli proteins allow reversal of the direction of rotation of the flagella in response to specific stimuli (Sockett et al., 1992; Welch et al., 1993). The hook connects the filament to the motor protein in the base. The helical filament is composed of many copies of the protein flagellin, and it can rotate clockwise (CW) and counterclockwise (CCW).

#### Directed motion: bacterial taxis

The term “taxis” refers to the movement toward or away from stimuli; it allows the bacteria to move toward suitable living environments. Many bacteria swim, propelled by rotation of the flagella outside the cell body. In contrast to eukaryotic flagella, bacteria flagella are rotors and—irrespective of species and type of flagellation—they only have two modes of operation: CW or CCW rotation. Bacterial swimming is used in bacterial taxis (mediated by specific receptors and signal transduction pathways) for the bacterium to move in a directed manner along gradients and reach more favorable conditions for life (Sowa and Berry, 2008; Krell et al., 2011). The direction of flagellar rotation is controlled by the type of molecules detected by the receptors on the surface of the cell: in the presence of an attractant gradient, the rate of smooth swimming increases, while the presence of a repellent gradient increases the rate of tumbling. For example, in *E. coli*, CCW rotation results in steady motion and CW rotation in tumbling; CCW rotation in a given direction is maintained longer in the presence of molecules of interest (like sugars or aminoacids) (Berg, 2004).

Depending on the stimulus that controls the directed movement, one can distinguish between chemotaxis (chemical gradients, e.g., glucose), aerotaxis (oxygen), pH-taxis, phototaxis, thermotaxis, and magnetotaxis. Bacterial taxis has been exploited for the directed movement of bacterial microswimmers. For example, a concentration gradient of L-serine was created with a micropipette and used as an attractant for *V. alginolyticus* carrying a 3 μm microbead (Nogawa et al., 2010). Similarly, *S. marcescens* cells attached to polystyrene microbeads of different sizes (5, 10, and 20 μm diameter) exhibited a clear indication of directional movement in the presence of L-aspartate as a chemoattractant (Kim et al., 2012). The pH-taxis of *S. marcescens* attached to 3 μm polystyrene beads has also been investigated (Zhuang et al., 2015). Magneto-aerotactic migration of *M. marinus* MC-1 was used to transport drug-loaded nanoliposomes into tumor hypoxic regions (Felfoul et al., 2016). Magnetotactic bacteria have been the focus of different studies due to their versatile uses in biohybrid motion systems (Lu and Martel, 2006; Faivre and Schüler, 2008; Martel, 2012; Taherkhani et al., 2014; Klumpp et al., 2017). Their unique properties are very complex and require separate consideration; therefore, they are not presented in detail in this work.

#### Swimming

The archetype of bacterial swimming is represented by the well-studied model organism *E. coli:* with its peritrichous flagellation, *E. coli* performs a run-and-tumble swimming pattern (Figure 2D). CCW rotation of the flagellar motors leads to flagellar bundle formation that pushes the cell in a forward run, parallel to the long axis of the cell. CW rotation disassembles the bundle and the cell rotates randomly (tumbling). After the tumbling event, straight swimming is recovered in a new direction (Berg, 2004). However, the type of swimming movement (propelled by rotation of flagella outside the cell body) varies significantly with the species and number/distribution of flagella on the cell body (Figure 2D). For example, the marine bacterium *V. alginolyticus*, with its single polar flagellum, swims in a cyclic, three-step (forward, reverse, and flick) pattern (Figure 2D). Forward swimming occurs when the flagellum pushes the cell head, while backward swimming is based on the flagellum pulling the head upon motor reversal. Besides these 180° reversals, the cells can reorient (a “flick”) by an angle around 90°, referred to as turning by buckling (Xie et al., 2011; Son et al., 2013). *Rhodobacter sphaeroides* with its subpolar monotrichous flagellation, represents yet another motility strategy (Armitage and Macnab, 1987; Haya et al., 2011): the flagellum only rotates in one direction, and it stops and coils against the cell body from time to time, leading to cell body reorientations (Figure 2D; Armitage et al., 1999; Pilizota et al., 2009; Rosser et al., 2014). In the soil bacterium *Pseudomonas putida*, a tuft of helical flagella is attached to its posterior pole. *P. putida* alternates between three swimming modes: pushing, pulling, and wrapping (Figure 2D; Hintsche et al., 2017). In the pushing mode, the rotating flagella (assembled in a bundle or as an open tuft of individual filaments) drive the motion from the rear end of the cell body. The trajectories are either straight or, in the vicinity of a solid surface, curved to the right, due to hydrodynamic interaction of the cell with the surface. The direction of curvature indicates that pushers are driven by a left-handed helix turning in CCW direction. In the pulling mode, the rotating flagellar bundle is pointing ahead. In this case the trajectories are either straight or with a tendency to bend to the left, indicating that pullers swim by turning a left-handed helical bundle in CW direction. Finally, *P. putida* can swim by wrapping the filament bundle around its cell body, with the posterior pole pointing in the direction of motion. In that case, the flagellar bundle takes the form of a left-handed helix that turns in CW direction, and the trajectories are predominantly straight (Hintsche et al., 2017).

Swimming bacterial cells have been used in the development of hybrid microswimmers (Di Leonardo et al., 2010; Zhang et al., 2013; Stanton et al., 2016; Suh et al., 2016). It should be noted that cargo attachment to the bacterial cells might influence their swimming behavior.

The hydrodynamics of bacterial swimming have been recently reviewed (Lauga, 2016) and will not be described here.

#### Swarming

The transition from swimming to swarming mobility is usually associated with an increase in the number of flagella per cell, accompanied by cell elongation (Kearns, 2010). Experiments with *Proteus mirabilis* showed that swarming requires contact between cells: swarming cells move in side-by-side groups called rafts, which dynamically add or lose cells: when a cell is left behind the raft, its movement stops after a short time; when a group of cells moving in a raft make contact with a stationary cell, it is reactivated and incorporated into the raft (Morrison and Scott, 1966). More recently, Swiecicki and coworkers designed a polymer microfluidic system to confine *E. coli* cells in a quasi-two-dimensional layer of motility buffer in order to study different behaviors of cells transitioning from swimming to swarming movement (Swiecicki et al., 2013). For this, they forced *E. coli* planktonic cells into a swarming-cell-phenotype by inhibiting cell division (leading to cell elongation) and by deletion of the chemosensory system (leading to smooth swimming cells that do not tumble). The increase of bacterial density inside the channel led to the formation of progressively larger rafts. Cells colliding with the raft contributed to increase its size, while cells moving at a velocity different from the mean velocity within the raft separated from it (Swiecicki et al., 2013).

Cell trajectories and flagellar motion during swarming was thoroughly studied for *E. coli*, in combination with fluorescently labeled flagella (Turner et al., 2000, 2010). The authors described four different types of tracks during bacterial swarming: forward movement, reversals, lateral movement, and stalls (Turner et al., 2010). In forward movement, the long axis of the cell, the flagellar bundle and the direction of movement are aligned, and propulsion is similar to the propulsion of a freely swimming cell. In a reversal, the flagellar bundle loosens, with the filaments in the bundle changing from their “normal form” (left-handed helices) into a “curly” form of right-handed helices with lower pitch and amplitude. Without changing its orientation, the cell body moves backwards through the loosened bundle. The bundle re-forms from curly filaments on the opposite pole of the cell body, and the filaments eventually relax back into their normal form. Lateral motion can be caused by collisions with other cells or by a motor reversal. Finally, stalled cells are paused but the flagella continue spinning and pumping fluid in front of the swarm, usually at the swarm edge.

Bacterial cells in the swarming state have also been used in the development of hybrid microswimmers. Swarming *S. marcescens* cells were transferred to PDMS-coated coverslips, resulting in a structure referred to as a “bacterial carpet” by the authors. Differently shaped flat fragments of this bacterial carpets, termed “auto-mobile chips,” moved above the surface of the microscope slide in two dimensions (Darnton et al., 2004). Many other works have used *S. marcescens* swarming cells (Behkam and Sitti, 2006; Steager et al., 2007, 2011; Park et al., 2010; Traoré et al., 2011; Kim and Kim, 2016), as well as *E. coli* swarming cells (Singh and Sitti, 2016; Park et al., 2017) for the development of hybrid microswimmers.

## Binding strategies for the preparation of bacteriabots

Bacterial attachment to the inorganic constituent is a critical stage in the preparation of BacteriaBots, dependent on the physical and surface chemistry of both components (Barroso et al., 2015; Wang and Pumera, 2018).

It is known that interactions of bacteria with surfaces produce changes in the expression of genes that influence their morphology and behavior. Moreover, attachment to surfaces can lead to advantageous features for the bacteria, such as biofilm formation, increased antibiotic resistance, and facilitated swarming behavior (Tuson and Weibel, 2013). In the initial instantaneous and reversible stage of attachment to surfaces, a combination of hydrodynamic and electrostatic interactions take place. Some physicochemical effects for bacteria during this stage include the loss of interfacial water and structural changes of surface molecules (Busscher et al., 2010; Tuson and Weibel, 2013). In the second, non-reversible stage, the attachment takes place through van der Waals interactions between the surface and components present in the bacteria wall (adhesins, lipopolysaccharides), as well as between the surface and extracellular organelles involved in surface attachment (flagella, pili, and curli fibers) (Van Houdt and Michiels, 2005; Tuson and Weibel, 2013).

**Figure 3:**
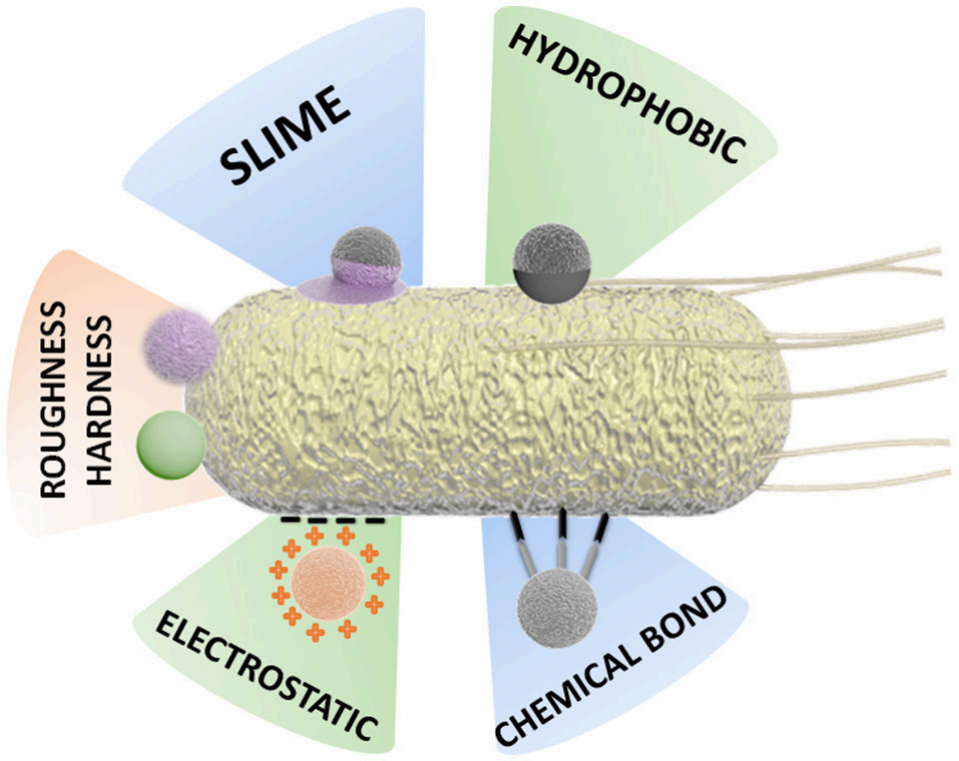
Schematic representation of different attachment strategies for the preparation of BacteriaBots.

This complex scenario of interaction between motile bacteria cells and the inorganic components of a BacteriaBot has stimulated the development of various binding strategies and preparation methodologies, as schematized in Figure 3.

Some binding methodologies are based mainly on physical attachment, for instance, by hydrophobic interaction. Other approaches are based on chemical interactions, for example, the formation of covalent bonds or binding by specific chemical attachments (streptavidin-biotin) (Tuson and Weibel, 2013; Hosseinidoust et al., 2016).

### Attachment strategies via physical synergies

#### Hydrophobic interaction

Since Gram-negative bacteria have many hydrophobic lipids in their outer surfaces, their surface energy can be lowered by attaching to hydrophobic objects, compared to the surface energy when surrounded by water during the free swimming state.

A strategy using microfluidics for anisotropic attachment of bacteria to polycaprolactone microcubes was proposed by Huh et al. (2015). The hydrophobicity of the biodegradable microcubic structures leads to fast bacterial adhesion with no further chemical modification or toxic adhesive required. Microfluidic channel systems have many advantages for the design of biohybrid microstructures, such as small sample volume, reduced reaction times and higher repeatability. Polycaprolactone microcubic structures (30 or 50 μm edge) were prepared using X-Ray synchrotron radiation and injected from the inlet reservoir into one straight channel device (width 2–2.5 mm) with chevron trappers separated from each other as a function of the size of the microcubes. Then, *S. typhimurium* bacteria were injected, and they anisotropically attached to the microcubic surface in less than 5s. The adhesion of bacteria was characterized by evaluating fluorescent intensity, in which the intensity of the two surfaces facing against the flow of bacteria is higher, as bacteria adsorption occurs only on them. The chemotactic velocity of the resulting BacteriaBot was measured by concentration gradient of aspartate, yielding average values of 5 μm/s.

#### Electrostatic interactions

As explained in section bacterial biohybrid microswimmers, the surface of bacteria is negatively charged, and hence positively charged objects offer a suitable surface for favorable attachment. This can be observed in a different patterning approach for the preparation of BacteriaBots concerning the preferential adhesion of *S. typhimurium* to poly-L-lysine (PLL). PLL was used to coat poly (ethylene glycol) (PEG) microbeads. Due to the submerging property of PEG beads on agarose gel, selective (half and total) coating with PLL was achieved. Characterization with Confocal Laser Scanning Microscopy using fluorescein labeled PLL revealed the patterned adhesion of bacteria on PEG microbeads, corresponding to the extent of coating with PLL (half or total) (Cho et al., 2012). It is likely that the electrostatic interaction between negatively charged lipopolysaccharide groups of the bacteria surface and the positively charged PLL is responsible for the selective bacteria adhesion (Rozhok et al., 2005).

An alternative bacteria-adhesive polymer was used by Stanton et al. in order to capture a single *E. coli* bacterium within a microtube internally modified with polydopamine (PDA) (Stanton et al., 2017a). In this case, the size ratio between bacteria and PDA microtubes influences swimming efficiency, and the design also includes a trigger mechanism in the tube to inhibit the swimming. Bacteria adhere to PDA without compromising viability and with great stability (even after exposure to solvents), making PDA a highly suitable surface for bacteria attachment. The attractive electrostatic interaction between the bacterium and PDA resulted in permanent attachment of the bacterium to the inside of the microtube while no attachment was observed in the outer region of the microtube.

#### Attachment by inherent response

Behkam and Sitti were the first to report the attachment of several *S*. *marcescens* bacteria to polystyrene (PS) microbeads (Behkam and Sitti, 2008). *S*. *marcescens* was used due to its relatively high speed and high density attachment to both hydrophobic (unpatterned) and hydrophilic (patterned) PS surfaces. As an intrinsic property of *S. marcescens*, the production of a sticky pink slime leads to relatively unselective adhesion to surfaces, which is the reason why a large number of biohybrid swimmers use this specific bacterium. As will be discussed later, adhesion can be suppressed by covering the surface of the artificial objects with surfactants.

In addition to this, Stanton and co-workers presented the use of Janus particles for the preparation of BacteriaBots in a one-step methodology, due to the preferential adhesion of *E. coli* on the metal (Au, Pt, Fe, Ti) capped face of particles with a PS or silica core. The favored attachment to the metal part was found to be related to its hydrophobicity. Attachment assays and contact angle and zeta potential characterization showed that *E. coli* prefer Pt surfaces over the other three metals investigated. The metal-free side of the particle can be further functionalized with a desirable surface chemistry (for instance, with the anticancer agent doxorubicin hydrochloride), showing the feasibility of the biohybrid for localized drug attachment (Stanton et al., 2016; Katuri et al., 2017).

Recently, Park et al. reported the preferential attachment of bacteria to stiffer elastic surfaces in comparison with softer surfaces, which could make the viscoelastic properties of the bacterial–surface interaction the most critical factor for bacterial attachment and the motility of biohybrid microswimmers (Park et al., 2017). This was achieved by embedding polyelectrolyte multilayer (PEM) nanoshell layers on PS microparticles (used as a core) resulting in a surface with feasible stiffness customization. Moreover, this approach allows control over the microparticle surface charge and chemistry. Specifically, PEM particles were synthesized by a layer-by-layer methodology (Decher, 1997). Thus, a single *E. coli* was attached to PEM that encapsulated doxorubicin (DOX) and magnetite (Fe_3_O_4_) nanoparticles. The resulting BacteriaBot exhibited directional motion in response to chemoattractant gradients and magnetic fields.

#### Attachment strategies via chemical interaction

One of the most commonly used chemical strategies for the preparation of BacteriaBots harnesses the high affinity interaction between biotin and streptavidin. Streptavidin is composed of four identical subunits; each subunit can bind a single biotin molecule (Wilchek et al., 2006). This protein and its derivatives have high thermal stability and resistance against extreme pH. Most streptavidin-based applications require chemical biotinylation of the target molecules (Dundas et al., 2013). Biotin is a small molecule present in living organisms for which covalent interaction with many proteins (such as streptavidin) can take place (Park et al., 2013b). Biotin/streptavidin conjugation does not imply a loss of biological activity. Accordingly, Gram-negative bacteria have been genetically engineered to display biotin in the outer membrane proteins all over the bacteria surface (Park et al., 2013b). This technique is an extensive tool in the fields of material functionalization, cell biology, and proteomics, as biotin/streptavidin (and derivatives) can be chemically modified (Ren et al., 2015). Park et al. used a motile strain of *S. typhimurium* to attach bacteria covalently to streptavidin-conjugated tandem fluorochrome of peridinin chlorophyll protein (PerCP), labeled with Cyanine (Cy5.5)-coated polystyrene microbeads, taking advantage of the high-affinity interaction between biotin and streptavidin.

Traore et al. described the assembly of *E. coli* with PS nanoparticles by the formation of a biotin/streptavidin complex between the streptavidin coated nanoparticles and bacteria (coated with biotin-conjugated antibody). The used antibody was raised against the O-antigens present on the cell membrane. This limits the attachment of the biotin-conjugated antibody (and thus streptavidin coated nanoparticles) mainly to the surface of the bacteria (Traore et al., 2014).

An extended approach obtains the linking of a bacteria to a liposome by means of a biotin-modified antibody, biotin-modified lipids, and streptavidin (Kojima et al., 2012). The study describes the effect on the motility of the biohybrid system when bacteria are directly attached to the liposome, and when they are attached via antibody linking. A so-called Raft Domain Binding Method controls the location of biotin on the liposome (and therefore the attachment position of bacteria), which is crucial for the evaluation of the biohybrid. A *V. alginolyticus* mutant strain was used for the fabrication of these BacteriaBots. A speed analysis of raft domain liposomes and normal liposomes driven by bacteria was screened by optical microscopy, and showed that the former move faster than the latter, due to the patterned assembly of bacteria.

A bioconjugation method based on a carbodiimide dehydrating agent was described for the covalent binding of terminal amine functionalities to carboxylated liposomes, without sacrificing motility and functionality. In the outer membrane of magnetotactic *M. marinus*, amine groups are present in different molecule types (phospholipids, lipopolysaccharides, lipoproteins, and polypeptides) (Taherkhani et al., 2014). This cross-linking approach is suitable for the preparation of conjugate biomolecules onto nano/microparticles with a stable chemical bond, which has relatively high enthalpy range values for amide bond formation (375–422 kJ mol^−1^) and high bond dissociation (305–440 kJ mol^−1^), in comparison with bioconjugation approaches based on van der Waals forces, electrostatic interaction, hydrogen bonds and hydrophobic effects (Luo, 2007). In addition to the strong bond generated by this methodology, the sample preparation is simple, highly reproducible, and is available at low cost. On the other hand, the effect of the chemical modification of the outer bacterial surface has not been studied in detail and might negatively influence the viability and resistance of bacteria.

## Effects of the shape of the artificial objects and obtained speeds

In general, the interactions of biological entities (cells, macrophages, bacteria…) with differently shaped objects is not sufficiently understood. It is well documented that particle size influences most aspects of interactions with living entities, including their degradability, attachment, uptake mechanisms, properties in flows, and excretion mechanisms. However, the lack of simple and reproducible fabrication methods for differently shaped nano- or microparticles makes studies relatively difficult. While some of the most extensive studies of interactions between eukaryotic cells and inorganic particles (Mathaes et al., 2013, 2014) find that uptake of elongated particles is lower compared to their spherical equivalents, this data only refers to one specific material, and these findings do not apply to bacteria. However, this topic has been treated in several excellent reviews (Tao et al., 2011; Champion et al., 2014; Jindal, 2017), and shall not be further considered here.

**Figure 4:**
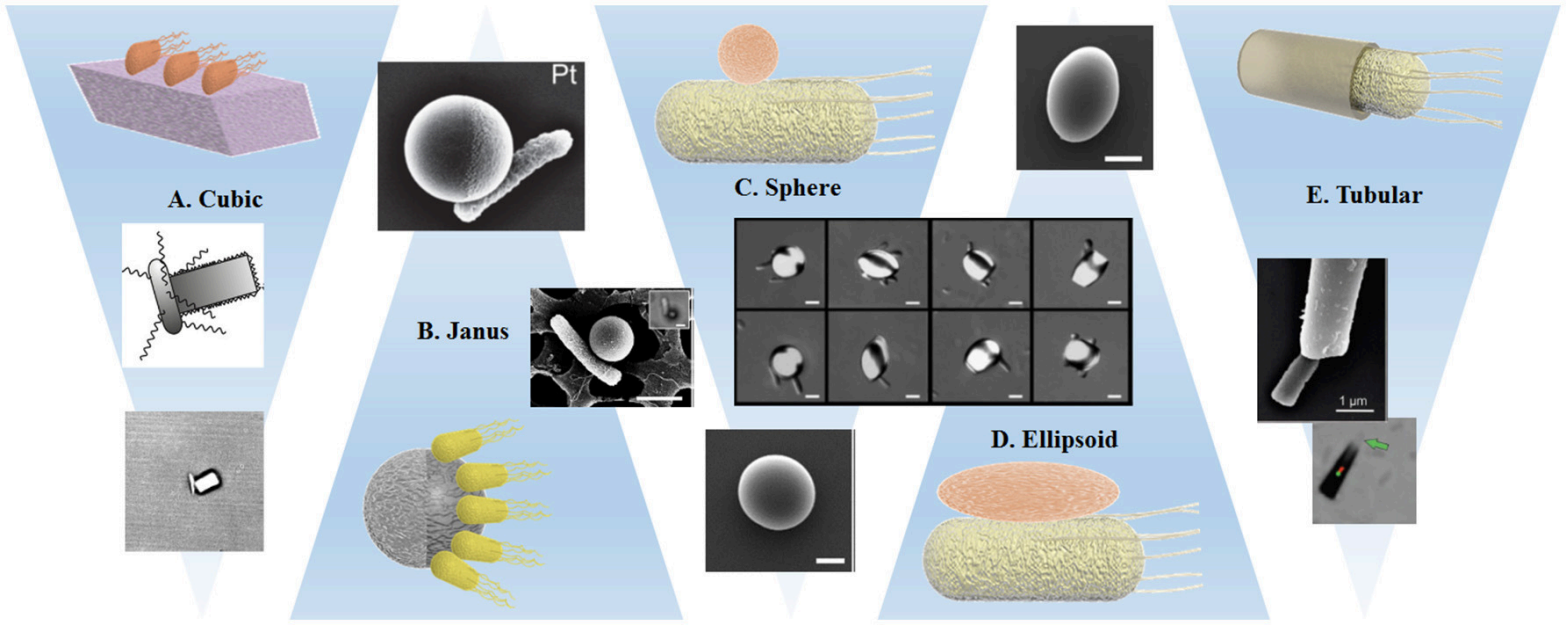
Schematic representations and examples of the interaction of bacteria with variously shaped cargoes: **(A)** Single bacterium attached by optical trapping to a cuboid elongated zeolite L crystal (Barroso et al., 2015). **(B,C)** Preferential attachment of bacteria to spherical metal capped-PS Janus particles (Stanton et al., 2016) and Pluronic covered PS particles (Behkam and Sitti, 2008). **(D)** Bacteria attached to PS particles with different ellipsoidal body geometries (Sahari et al., 2012). **(E)** Bacteria captured inside a microtube regarding the bacteria driven microswimmer assembly (Stanton et al., 2017a). All images presented with corresponding copyright permissions.

The relative scarcity of differently shaped particles has also affected the field of bacterial biohybrids, and few studies present experiments using the same bacterial strain and comparable materials, selected examples are presented in Figure 4. Before grouping and discussing the different approaches, we consider general conditions that most likely influence the dynamics at the microscale, owing to the domination of viscous forces over inertia. Even though the disturbances caused by the cargo objects in low Reynolds number laminar flow vanish quickly (in the sense that they leave no wake in the surrounding flow), the viscous forces exerted by the flow on the object strongly depend on the body geometry.

In one of the few articles targeting explicitly the influence of the shape of the artificial object, Sahari et al. compare the attachment of *E. coli* via a poly-L-lysine layer to PS microparticles, employing not only the initial spherical particles, but also ellipsoids, barrels, and prolate spheroids (Sahari et al., 2012) that have been obtained from the initial spheres by embedding them into a film and subsequent stretching. Since the size of the particle was about five times the bacteria size and up to six bacteria were attached to each microparticle, there was no significant effect on the speed, but the directionality of the BacterialMicrosystem was increased for elongated particle shapes in comparison to spheres.

The geometrical restriction of bacterial attachment can be achieved through different strategies: the earliest approach led to a BacterialMicrosystem and was presented by Behkam and Sitti. As mentioned in section Attachment Strategies via Physical Synergies, *S. marcescens* attach unselectively to surfaces, but avoid areas that are covered with a certain surfactant molecule (Pluronic®) (Behkam and Sitti, 2008). The authors treated Pluronic covered PS beads with a plasma to achieve a covered and a bare hemisphere and studied the attachment of the bacteria to the Pluronic free hemisphere. They found that the speed is ~28 μm/s for the patterned beads, compared to 14 μm/s for totally bacteria covered spheres. This trend can be explained by the cancelation of propulsion forces generated by attached bacteria on opposite sides of the beads, which is largely suppressed by patterning. A correlation of the speed of patterned beads with the number of attached bacteria distinguished the individual contribution of each bacterium to the dynamics of the motile system.

A BacteriaBot based on the attachment of *E. coli* to the metal caps of Janus particles also relied on hemispheres with different material properties (see Figure 4). However, due to much smaller bead sizes, this work obtained attachment of single bacteria, which enables a clear observation of the decrease of swimming speed after cargo attachment (Stanton et al., 2016). An isolated example of a cuboid amino functionalized zeolite cargo attached to *B. subtilis* by electrostatic interaction showed a similar decrease in swimming speed (Figure 4). The attachment probability was increased by placing the Zeolite in close proximity of the bacterium using an optical trap. Additionally, the authors observed a strong difference in swimming trajectories after cargo binding: curved trajectories were explained by reference to the asymmetric configuration of the BacteriaBot (Barroso et al., 2015).

As discussed previously, different binding approaches have been used on several materials of spherical shape (Hosseinidoust et al., 2016; Park et al., 2017), including liposomes (Kojima et al., 2013) without any consideration of the effect of shape. An approach based on a soft double microemulsion enabled the authors to obtain bacterial hybrids (mostly 1-2 bacteria per droplet) that can deform if pushed through narrow channels (Singh et al., 2017). However, this deformation was only observed in confined environments, and a characterization of the effect of shape independent from the confined environment was not possible. In an approach of Akin et al. quasi spherical streptavidin-coated polystyrene nanoparticles were bound to biotinylated monoclonal antibodies and via surface proteins to *L. monocytogenes* bacteria. Since remaining streptavidin sites on the nanoparticles were occupied by biotinylated GFP, plasmid imaging was facilitated (Akin et al., 2007). Subsequently, the authors showed that when these bacteria were able to enter the cells, the size and not the concrete shape of the inorganic part of the bacterial hybrid was responsible for the effortless passage through the cell membrane.

A strategy established by the Sanchez' group used different conical or tubular structures for a “binding” method that is based on trapping rather than attachment, since the bacteria swim inside the structure, where they are able to keep moving forward, but cannot reorient to swim out. The authors argue similarly to Barroso et al. (2015) that a particle attached to the bacterial body induces torques and rotational motion and therefore decreases the net translational motion, for which the conical structures offer an elegant solution. Additionally, the tubes seemed to suppress run and tumble behaviors; all microtube BacteriaBots displayed more steady and directional trajectories than their freely swimming counterparts. The microtubes can be obtained via electrodeposition (Stanton et al., 2017a) or a template based precipitation method resulting in silica microtubes internally functionalized with amino groups (Stanton et al., 2017b), and were tuned in size to match the bacteria size in both diameter and length in order to improve swimming efficiency. For the silica tubes, two different lengths were fabricated and slower speeds were obtained for the longer tubes, which was counterbalanced by increased drug loading capabilities (Stanton et al., 2017b).

A very different shape that also aims for bacterial trapping was fabricated by the group of DiLeonardo (Vizsnyiczai et al., 2017). Their optimized gear-like wheel structure, equipped with ramps for directing bacteria, likewise relies on the fact that *E. coli* cannot “swim backwards.” The elegant geometry consists of an outer circle of structures that align bacteria to swim toward a central gear-like structure. After passing through the outer circle, bacteria swim into microchambers on the rim of the gear that are optimized for capture of individual bacteria. The trapped bacteria continuously exert a torque on the gear. Even though this approach is driven by a large number of bacteria, each of them acts individually on the gear, and no effect of collective turbulence is observed. Several other approaches involving gear-like structures have been discussed in the literature. In all of these works, each micro-object is propelled by a large number of bacteria, but two general approaches have to be distinguished: in the first method, similarly to the surface patterned beads (Behkam and Sitti, 2008), structures of various sizes and shapes can be obtained and surface patterned with bacteria (here mostly *S. marcescens*). These are attached to the structures and move the surrounding fluid, resulting in effective propulsion. In the second case, the movement of a microobject is caused by bacterial turbulence, with no concrete binding of the bacteria to the surface of the structure.

Firstly, we want to briefly examine the direct attachment of so called “bacterial carpets” to microstructures. In an early work, Darnton et al. created active bacterial carpets to move micro-objects with the shape of beads, microcars, and wedges (Darnton et al., 2004). The authors tested different bacterial strains and found that only *S. marcescens* formed good bacterial carpets, while *E. coli* failed to bind completely, and *S. typhimurium* was little better. Even though the influence of shape was not discussed in depth, the authors characterized the position of the flagella above the carpets. In addition, the flows created by the bacteria—which were responsible for motion of the objects—were characterized by adding tracer particles. In another work, Kim & Kim relied on the slime produced by *S. marcescens*, which adheres naturally to surfaces, to create a bacterial carpet on SU8 squares (Kim et al., 2014; Kim and Kim, 2016). The collective motion of the flagella actuates the BacterialMicrostructure. The negative surface charges of the bacteria enable the whole BacterialMicrostructure to follow an electric field, which offers an elegant guiding mechanism when coupled to a feedback loop. A similar approach of control by electric fields was followed by Steager et al. (2011), and later on further investigated to elucidate the mechanism of motion and the observed prevalence of clockwise motion (Wong et al., 2014). By comparing squares and gear structures, the authors found that bacteria that adhered to the surface walls of large microstructures were influenced much less by the shape of the structure than expected, at least for the large microstructure/bacterium size ratio that was considered.

The second strategy to move micro-objects does not rely on strict binding of the bacteria to the object surface, but rather achieves effective propulsion by bacterial turbulence, causing significant differences not only in the steadiness of the motion, but also in theoretical aspects and in versatility of the approach. Here, the most well-known examples include experiments on gear shaped micro-objects (Sokolov et al., 2009; Di Leonardo et al., 2010), where momentum is transferred to the gears by collective swimming and collisions with the microstructures. In these systems, the shape of the object is of pivotal importance, since it controls the force transduction from the bacterial motion to propulsion of the passive objects. However, DiLeonardo et al. presented a different experimental setup where bacterial turbulences were able to move spherical, symmetric beads, and directionality was achieved by 3D-structuration of the environment (Koumakis et al., 2013).

In summary, it can be said that the influence of shape on the features of a bacterial biohybrid microswimmer is very poorly analyzed and understood, and more systematic evaluation, categorized after bacterial type, swimming strategy, and binding modality, are needed to deduce clear effects of the object shape.

## Applications, challenges, and outlook

There are different potential applications of bacterial biohybrid microswimmers that exploit their capability to transport cargoes or reach targets in a customized way. For instance, these hybrid bioswimmers have been tested as drug delivery systems (Wang and Pumera, 2018). Thus, the combination of bacteria with abiotic systems (e.g., micro particles, liposomes) leads to advanced levels of functionalization not reachable by each component separately. For example, the capability of bacteria to sense and response to environmental stimuli can be harnessed to make them capable of looking for specific biomarkers and to accumulate at targeted locations in the body. Thus, the *in situ* production or selective release of therapeutic agents using bacterial biohybrid microswimmers would lead to reductions in the cost of therapy and the need for purification of drugs produced *ex situ*, as well as to a significant decrease of the dose of medicament supplied (Hosseinidoust et al., 2016). For instance, Mostaghaci et al. accomplished the release of a drug at the site of a disease by a bioadhesive approach, and therefore increased the efficiency of drug delivery (Mostaghaci et al., 2017). Anchoring BacteriaBots to epithelial cells was achieved by bonding lectin molecules (from the tip of type 1 pili) to mannose molecules present in the cell. This approach could be extended to more precise targeting functionalities as antibodies or aptamers, or alternatively used for the attachment of particles to bacteria.

Furthermore, the individual and combined power of bacterial movement can be exploited by the creation of hybrid micromachines for the development of mechanical tasks and applications. For example, the chemotactic properties of *E. coli* were exploited to sort and separate similarly-sized nanoparticles of dissimilar surface properties (Suh et al., 2016). *P. mirabilis* swimming bacteria were used to push non-motile *Candida albicans* fungal cells (Trivedi et al., 2015). Taking magnetotactic bacteria under consideration, Stanton et al. showed that the magnetic guidability of the system can be used to direct BacteriaBots toward an *E. coli* biofilm. The biofilm could be attacked with antibiotics and was therefore more susceptible to chemical threat, even though the biofilm was resistant to conventional treatment (Stanton et al., 2017b). This result demonstrates that an agile strategy for drug delivery using bacterial biohybrid microswimmers can bring new advantages in tackling even notoriously thorny problems like multi resistant germs. Additionally, the delivery of pharmaceutics and nutrients within the body could benefit from faster delivery through the bacteria naturally present in the microbiome of human and animal bodies. Since many modern medicines are produced by transgenic bacteria, the idea to improve the production of insulin or other proteins within the bacteria through direct combination with *in situ* encapsulation of the protein into the cargo structure no longer seems so much like science fiction. However, the potential of bacterial hybrid swimmers is not limited to biomedical applications; the inherent sensitivity of bacteria to environmental stimuli could be well exploited to create motile sensors. This could build on the signal transduction systems inherent to bacteria, which allow the creation of whole-cell biosensors used, for example, in antibiotic discovery (Wolf and Mascher, 2016). These signal transduction pathways are maintained when objects are added on the surface and might eliminate sample preparation steps in current analytical procedures, or pave the ways for completely new approaches.

However, a series of challenges still has to be addressed before the large scale use and production of bacterial biohybrids: to start with, it has yet to be shown that selective attachment can occur even in protein rich natural environments, since most binding strategies have only been applied *in vitro*. Ideally, after cell division, daughter cells should bind immediately and automatically to available cargos in order to avoid “dilution” of the BacteriaBots. Finally, the detachment, and release of the cargo still has to be engineered.

Concluding, we can say that the field of bacterial hybrid swimmers opens many promising ways to couple custom adaptable cargos to one of evolution's masterpieces, the bacterium. Even today, bacteria play a major role in modern biotech, and we dare to predict that the bacterial biohybrids will increase this even further.

## Author contributions

All authors listed have made a substantial, direct and intellectual contribution to the work, and approved it for publication.

### Conflict of interest statement

The authors declare that the research was conducted in the absence of any commercial or financial relationships that could be construed as a potential conflict of interest. The reviewer, AK, and handling Editor declared their shared affiliation.

## References

[B1] Akin D. Sturgis J. Ragheb K. Sherman D. Burkholder K. Robinson J. P.. (2007). Bacteria-mediated delivery of nanoparticles and cargo into cells. Nat. Nanotechnol. 2, 441–449. 10.1038/nnano.2007.149 18654330

[B2] Armitage J. P. Macnab R. M. (1987). Unidirectional, intermittent rotation of the flagellum of *Rhodobacter sphaeroides*. J. Bacteriol. 169, 514–518. 10.1128/jb.169.2.514-518.1987 3492489 PMC211807

[B3] Armitage J. P. Pitta T. P. Vigeant M. A. Packer H. L. Ford R. M. (1999). Transformations in flagellar structure of *Rhodobacter sphaeroides* and possible relationship to changes in swimming speed. J. Bacteriol. 181, 4825–4833. 10438751 10.1128/jb.181.16.4825-4833.1999 PMC93968

[B4] Asano T. Ishizua T. Yawo H. (2012). Optically controlled contraction of photosensitive skeletal muscle cells. Biotechnol. Bioeng. 109, 199–204. 10.1002/bit.23285 21809334

[B5] Barroso Á. Landwerth S. Woerdemann M. Alpmann C. Buscher T. Becker M.. (2015). Optical assembly of bio-hybrid micro-robots. Biomed. Microdevices 17, 1–8. 10.1007/s10544-015-9933-1 25681045 PMC4328111

[B6] Beeby M. Gumbart J. C. Roux B. Jensen G. J. (2013). Architecture and assembly of the Gram-positive cell wall. Mol. Microbiol. 88, 664–672. 10.1111/mmi.12203 23600697 PMC3663049

[B7] Behkam B. Sitti M. (2006). Towards hybrid swimming microrobots: bacteria assisted propulsion of polystyrene beads, in Annual International Conference of the IEEE Engineering in Medicine and Biology - Proceedings (New York, NY), 2421–2424. 10.1109/IEMBS.2006.259841 17946113

[B8] Behkam B. Sitti M. (2008). Effect of quantity and configuration of attached bacteria on bacterial propulsion of microbeads. Appl. Phys. Lett. 93:223901 10.1063/1.3040318

[B9] Berg H. (2004). E. coli in Motion. Cambridge, MA: Springer.

[B10] Berg H. C. (2003). The Rotary motor of bacterial flagella. Annu. Rev. Biochem. 72, 19–54. 10.1146/annurev.biochem.72.121801.161737 12500982

[B11] Berg H. C. Anderson R. A. (1973). Bacteria swim by rotating their flagellar filaments. Nature 245, 380–382. 10.1038/245380a0 4593496

[B12] Braun V. (1975). Covalent lipoprotein from the outer membrane of *Escherichia coli*. BBA Rev. Biomembr. 415, 335–377. 10.1016/0304-4157(75)90013-1 52377

[B13] Brown R. (1828). XXVII. A brief account of microscopical observations made in the months of June, July and August 1827, on the particles contained in the pollen of plants; and on the general existence of active molecules in organic and inorganic bodies. Philos. Mag. 4, 161–173. 10.1080/14786445208647098

[B14] Busscher H. J. Norde W. Sharma P. K. van der Mei H. C. (2010). Interfacial re-arrangement in initial microbial adhesion to surfaces. Curr. Opin. Colloid Interface Sci. 15, 510–517. 10.1016/j.cocis.2010.05.014

[B15] Campuzano S. Orozco J. Kagan D. Guix M. Gao W. Sattayasamitsathit S.. (2012). Bacterial isolation by lectin-modified microengines. Nano Lett. 12, 396–401. 10.1021/nl203717q 22136558 PMC3256279

[B16] Carlsen R. W. Edwards M. R. Zhuang J. Pacoret C. Sitti M. (2014). Magnetic steering control of multi-cellular bio-hybrid microswimmers. Lab Chip 14, 3850–3859. 10.1039/C4LC00707G 25120224

[B17] Carlsen R. W. Sitti M. (2014). Bio-hybrid cell-based actuators for microsystems. Small 10, 3831–3851. 10.1002/smll.201400384 24895215

[B18] Champion J. A. Katare Y. K. Mitragotri S. (2014). Particle Shape: a new design parameter for micro- and nanoscale drug delivery carriers. J. Control. Release 121, 3–9. 10.1016/j.jconrel.2007.03.022 17544538 PMC4009069

[B19] Chen S. Beeby M. Murphy G. E. Leadbetter J. R. Hendrixson D. R. Briegel A.. (2011). Structural diversity of bacterial flagellar motors. EMBO J. 30, 2972–2981. 10.1038/emboj.2011.186 21673657 PMC3160247

[B20] Cho S. Park S. J. Ko S. Y. Park J. O. Park S. (2012). Development of bacteria-based microrobot using biocompatible poly(ethylene glycol). Biomed. Microdevices 14, 1019–1025. 10.1007/s10544-012-9704-1 22976580

[B21] Darnton N. Turner L. Breuer K. Berg H. C. (2004). Moving fluid with bacterial carpets. Biophys. J. 86, 1863–1870. 10.1016/S0006-3495(04)74253-8 14990512 PMC1304020

[B22] Decher G. (1997). Fuzzy nanoassemblies: toward layered polymeric multicomposites. Science 277, 1232–1237. 10.1126/science.277.5330.1232

[B23] Di Leonardo R. Angelani L. Dell'Arciprete D. Ruocco G. Iebba V. Schippa S.. (2010). Bacterial ratchet motors. Proc. Natl. Acad. Sci.U.S.A. 107, 9541–9545. 10.1073/pnas.0910426107 20457936 PMC2906854

[B24] Drescher K. Goldstein R. E. Tuval I. (2010). Fidelity of adaptive phototaxis. Proc. Natl. Acad. Sci.U.S.A. 107, 11171–11176. 10.1073/pnas.1000901107 20534560 PMC2895142

[B25] Dufrêne Y. F. (2015). Sticky microbes: forces in microbial cell adhesion. Trends Microbiol. 23, 376–382. 10.1016/j.tim.2015.01.011 25684261

[B26] Dundas C. M. Demonte D. Park S. (2013). Streptavidin-biotin technology: improvements and innovations in chemical and biological applications. Appl. Microbiol. Biotechnol. 97, 9343–9353. 10.1007/s00253-013-5232-z 24057405

[B27] Ehrmann M. Meltzer M. Kucz N. (2007). Periplasmic proteases and protease inhibitors, in The Periplasm, ed Ehrmann M. (Washington, DC: ASM Press), 150–170.

[B28] Einstein A. (1905). Über die von der molekularkinetischen Theorie der Wärme geforderte Bewegung von in ruhenden Flüssigkeiten suspendierten Teilchen. Ann. Phys. 322, 549–560. 10.1002/andp.19053220806

[B29] Elston T. C. Oster G. (1997). Protein turbines I: the bacterial flagellar motor. Biophys. J. 73, 703–721. 10.1016/S0006-3495(97)78104-9 9251788 PMC1180968

[B30] Erhardt M. Namba K. Hughes K. T. (2010). Bacterial nanomachines: the flagellum and type III injectisome. Cold Spring Harb. Perspect. Biol. 2:a000299. 10.1101/cshperspect.a000299 20926516 PMC2964186

[B31] Evans L. D. Hughes C. Fraser G. M. (2014). Building a flagellum outside the bacterial cell. Trends Microbiol. 22, 566–572. 10.1016/j.tim.2014.05.009 24973293 PMC4183434

[B32] Faivre D. Schüler D. (2008). Magnetotactic bacteria and magnetosomes. Chem. Rev. 108, 4875–4898. 10.1021/cr078258w 18855486

[B33] Felfoul O. Mohammadi M. Gaboury L. Martel S. (2011). Tumor targeting by computer controlled guidance of magnetotactic bacteria acting like autonomous microrobots, in IEEE International Conference on Intelligent Robots and Systems (San Francisco, CA) 1304–1308.

[B34] Felfoul O. Mohammadi M. Taherkhani S. De Lanauze D. Zhong Xu Y. Loghin D.. (2016). Magneto-aerotactic bacteria deliver drug-containing nanoliposomes to tumour hypoxic regions. Nat. Nanotechnol. 11, 941–947. 10.1038/nnano.2016.137 27525475 PMC6094936

[B35] Feringa B. L. (2001). In control of motion: from molecular switches to molecular motors. Acc. Chem. Res. 34, 504–513. 10.1021/ar0001721 11412087

[B36] Friedrich B. M. Riedel-Kruse I. H. Howard J. Jülicher F. (2010). High-precision tracking of sperm swimming fine structure provides strong test of resistive force theory. J. Exp. Biol. 213, 1226–1234. 10.1242/jeb.039800 20348333

[B37] Haya S. Tokumaru Y. Abe N. Kaneko J. Aizawa S. (2011). Characterization of lateral flagella of *Selenomonas ruminantium*. Appl. Environ. Microbiol. 77, 2799–2802. 10.1128/AEM.00286-11 21335384 PMC3126368

[B38] Henrichsen J. (1972). Bacterial surface translocation: a survey and a classification. Bacteriol. Rev. 36, 478–503. 4631369 10.1128/br.36.4.478-503.1972 PMC408329

[B39] Hintsche M. Waljor V. Großmann R. Kühn M. J. Thormann K. M. Peruani F.. (2017). A polar bundle of flagella can drive bacterial swimming by pushing, pulling, or coiling around the cell body. Sci. Rep. 7:16771. 10.1038/s41598-017-16428-9 29196650 PMC5711944

[B40] Hiratsuka Y. Miyata M. Tada T. Uyeda T. Q. (2006). A microrotary motor powered by bacteria. Proc. Natl. Acad. Sci.U.S.A. 103, 13618–13623. 10.1073/pnas.0604122103 16950878 PMC1564248

[B41] Hiratsuka Y. Miyata M. Uyeda T. Q. (2005). Living microtransporter by uni-directional gliding of Mycoplasma along microtracks. Biochem. Biophys. Res. Commun. 331, 318–324. 10.1016/j.bbrc.2005.03.168 15845395

[B42] Hirota N. Kitada M. Imae Y. (1981). Flagellar motors of *Alkalophilic bacillus* are powered by an electrochemical potential gradient of Na+. FEBS Lett. 132, 278–280. 10.1016/0014-5793(81)81178-7

[B43] Hölscher T. Kovács Á. T. (2017). Sliding on the surface: bacterial spreading without an active motor. Environ. Microbiol. 19, 2537–2545. 10.1111/1462-2920.13741 28370801

[B44] Hosseinidoust Z. Mostaghaci B. Yasa O. Park B. W. Singh A. V. Sitti M. (2016). Bioengineered and biohybrid bacteria-based systems for drug delivery. Adv. Drug Deliv. Rev. 106, 27–44. 10.1016/j.addr.2016.09.007 27641944

[B45] Huh K. Oh D. Yoo H. J. Song B. Cho D. I. D. Seo J. M. (2015). Bacteria-based microrobot for chemotaxis delivery, in ICCAS 2015 - 2015 15th International Conference on Control, Automation and Systems, Proceedings (Busan), 1848–1852.

[B46] Islam S. T. Mignot T. (2015). The mysterious nature of bacterial surface (gliding) motility: a focal adhesion-based mechanism in *Myxococcus xanthus*. Semin. Cell Dev. Biol. 46, 143–154. 10.1016/j.semcdb.2015.10.033 26520023

[B47] Ismagilov R. F. Schwartz A. Bowden N. Whitesides G. M. (2002). Autonomous movement and self-assembly. Angew. Chem. 114, 674–676. 10.1002/1521-3773(20020215)41:4<652::AID-ANIE652>3.0.CO;2-U

[B48] Jindal A. B. (2017). The e ff ect of particle shape on cellular interaction and drug delivery applications of micro-and nanoparticles. Int. J. Pharm. 532, 450–465. 10.1016/j.ijpharm.2017.09.028 28917985

[B49] Katuri J. Ma X. Stanton M. M. Sánchez S. (2017). Designing micro-and nanoswimmers for specific applications. Acc. Chem. Res. 50, 2–11. 10.1021/acs.accounts.6b00386 27809479 PMC5244436

[B50] Kearns D. B. (2010). A field guide to bacterial swarming motility. Nat. Rev. Microbiol. 8, 634–644. 10.1038/nrmicro2405 20694026 PMC3135019

[B51] Kim D. Liu A. Diller E. Sitti M. (2012). Chemotactic steering of bacteria propelled microbeads. Biomed. Microdevices 14, 1009–1017. 10.1007/s10544-012-9701-4 22960953

[B52] Kim H. Cheang U. K. Kim M. J. Lee K. (2014). Obstacle avoidance method for microbiorobots using electric field control, in Cyber Technology in Automation, Control, and Intelligent Systems (CYBER), 2014 IEEE 4th Annual International Conference on (IEEE) (Hong Kong), 117–122.

[B53] Kim H. Kim M. J. (2016). Electric field control of bacteria-powered microrobots using a static obstacle avoidance algorithm. IEEE Trans. Robot. 32, 125–137. 10.1109/TRO.2015.2504370

[B54] Klumpp S. Lefevre C. Landau L. Codutti A. Bennet M. Faivre D. (2017). Magneto-Aerotaxis: bacterial motility in magnetic fields. Biophys. J. 112:567a 10.1016/j.bpj.2016.11.3052

[B55] Kojima M. Zhang Z. Nakajima M. Fukuda T. (2012). High efficiency motility of bacteria-driven liposome with raft domain binding method. Biomed. Microdevices 14, 1027–1032. 10.1007/s10544-012-9711-2 23053448

[B56] Kojima M. Zhang Z. Nakajima M. Ooe K. Fukuda T. (2013). Construction and evaluation of bacteria-driven liposome. Sensors Actuat. B Chem. 183, 395–400. 10.1016/j.snb.2013.03.127

[B57] Koumakis N. Lepore A. Maggi C. Di Leonardo R. (2013). Targeted delivery of colloids by swimming bacteria. Nat. Commun. 4, 1–6. 10.1038/ncomms3588 24100868 PMC4112550

[B58] Krell T. Lacal J. Muñoz-Martínez F. Reyes-Darias J. A. Cadirci B. H. García-Fontana C.. (2011). Diversity at its best: bacterial taxis. Environ. Microbiol. 13, 1115–1124. 10.1111/j.1462-2920.2010.02383.x 21087385

[B59] Lauga E. (2016). Bacterial Hydrodynamics. Annu. Rev. Fluid Mech. 48, 105–130. 10.1146/annurev-fluid-122414-034606

[B60] Lauga E. Powers T. R. (2009). The hydrodynamics of swimming microorganisms. Rep. Prog. Phys. 72:096601 10.1088/0034-4885/72/9/096601

[B61] Leifson E. (1960). Atlas of Bacterial Flagellation. New York, NY: Academic Press 10.5962/bhl.title.7270

[B62] Li D. Choi H. Cho S. Jeong S. Jin Z. Lee C.. (2015). A hybrid actuated microrobot using an electromagnetic field and flagellated bacteria for tumor-targeting therapy. Biotechnol. Bioeng. 112, 1623–1631. 10.1002/bit.25555 25944679

[B63] Lindemann C. B. (2014). Engaging the “clutch” to move forward. Biophys. J. 107, 1487–1488. 10.1016/j.bpj.2014.07.065 25296298 PMC4190595

[B64] Lindemann C. B. Lesich K. A. (2010). Flagellar and ciliary beating: the proven and the possible. J. Cell Sci. 123, 519–528. 10.1242/jcs.051326 20145000

[B65] Lu Z. Martel S. (2006). Preliminary investigation of bio-carriers using magnetotactic bacteria, in Engineering in Medicine and Biology Society, 2006. EMBS'06. 28th Annual International Conference of the IEEE (New York, NY: IEEE), 3415–3418. 10.1109/IEMBS.2006.260299 17947027

[B66] Luo C. H. Huang C. T. Su C. H. Yeh C. S. (2016). Bacteria-mediated hypoxia-specific delivery of nanoparticles for tumors imaging and therapy. Nano Lett. 16, 3493–3499. 10.1021/acs.nanolett.6b00262 27148804

[B67] Luo Y. R. (2007). Comprehensive Handbook of Chemical Bond Energies. Boca Ratón, FL: CRC Press.

[B68] Madigan M. T. Bender K. S. Buckley D. H. Martinko J. M. Stahl D. A. (2014). Brock Biology of Microorganisms. Amsterdam: Addison-Wesley Longman.

[B69] Magdanz V. Medina-Sánchez M. Schwarz L. Xu H. Elgeti J. Schmidt O. G. (2017). Spermatozoa as functional components of robotic microswimmers. Adv. Mater. Weinheim. 29:1606301. 10.1002/adma.201606301 28323360

[B70] Magdanz V. Sanchez S. Schmidt O. G. (2013). Development of a sperm-flagella driven micro-bio-robot. Adv. Mater. Weinheim. 25, 6581–6588. 10.1002/adma.201302544 23996782

[B71] Manson M. D. Tedesco P. Berg H. C. Harold F. M. Van der Drift C. (1977). A protonmotive force drives bacterial flagella. Proc. Natl. Acad. Sci.U.S.A. 74, 3060–3064. 10.1073/pnas.74.7.3060 19741 PMC431412

[B72] Martel S. (2012). Bacterial microsystems and microrobots. Biomed. Microdevices 14, 1033–1045. 10.1007/s10544-012-9696-x 22960952

[B73] Mathaes R. Winter G. Besheer A. Engert J. (2014). Influence of particle geometry and PEGylation on phagocytosis of particulate carriers. Int. J. Pharm. 465, 159–164. 10.1016/j.ijpharm.2014.02.037 24560647

[B74] Mathaes R. Winter G. Engert J. Besheer A. (2013). Application of different analytical methods for the characterization of non-spherical micro- And nanoparticles. Int. J. Pharm. 453, 620–629. 10.1016/j.ijpharm.2013.05.046 23727141

[B75] Mattick J. S. (2002). Type IV Pili and Twitching Motility. Annu. Rev. Microbiol. 56, 289–314. 10.1146/annurev.micro.56.012302.160938 12142488

[B76] Medina-Sánchez M. Schwarz L. Meyer A. K. Hebenstreit F. Schmidt O. G. (2016). Cellular Cargo Delivery: toward assisted fertilization by sperm-carrying micromotors. Nano Lett. 16, 555–561. 10.1021/acs.nanolett.5b04221 26699202

[B77] Minamino T. Imada K. (2015). The bacterial flagellar motor and its structural diversity. Trends Microbiol. 23, 267–274. 10.1016/j.tim.2014.12.011 25613993

[B78] Montemagno C. Bachand G. (1999). Constructing nanomechanical devices powered by biomolecular motors. Nanotechnology 10, 225–231. 10.1088/0957-4484/10/3/301

[B79] Morrison R. B. Scott A. (1966). Swarming of proteus-A solution to an old problem? Nature 211, 255–257. 10.1038/211255a0 5965543

[B80] Mostaghaci B. Yasa O. Zhuang J. Sitti M. (2017). Bioadhesive bacterial microswimmers for targeted drug delivery in the urinary and gastrointestinal tracts. Adv. Sci. 4, 1–9. 10.1002/advs.201700058 28638787 PMC5473323

[B81] Nan B. Zusman D. R. (2016). Novel mechanisms power bacterial gliding motility. Mol. Microbiol. 101, 186–193. 10.1111/mmi.13389 27028358 PMC5008027

[B82] Neuhaus F. C. Baddiley J. (2003). A continuum of anionic charge: structures and functions of D-alanyl-teichoic acids in gram-positive bacteria. Microbiol. Mol. Biol. Rev. 67, 686–723. 10.1128/MMBR.67.4.686-723.2003 14665680 PMC309049

[B83] Nogawa K. Kojima M. Nakajima M. Homma M. Fukuda T. (2010). Motion control of bacteria-driven micro objects by Nano/Micro pipettes, in Nanotechnology (IEEE-NANO), 2010 10th IEEE Conference on (IEEE) (Seoul), 1028–1031.

[B84] Ozin G. A. Manners I. Fournier-Bidoz S. Arsenault A. (2005). Dream nanomachines. Adv. Mater. Weinheim. 17, 3011–3018. 10.1002/adma.200501767

[B85] Park B. W. Zhuang J. Yasa O. Sitti M. (2017). Multifunctional bacteria-driven microswimmers for targeted active drug delivery. ACS Nano 11, 8910–8923. 10.1021/acsnano.7b03207 28873304

[B86] Park S. J. Bae H. Kim J. Lim B. Park J. Park S.. (2010). Motility enhancement of bacteria actuated microstructures using selective bacteria adhesion. Lab Chip 10, 1706–1711. 10.1039/c000463d 20422075

[B87] Park S. J. Bae H. Ko S. Y. Min J. J. Park J. O. Park S. (2013a). Selective bacterial patterning using the submerged properties of microbeads on agarose gel. Biomed. Microdevices 15, 793–799. 10.1007/s10544-013-9765-9 23674143

[B88] Park S. J. Park S. H. Cho S. Kim D. M. Lee Y. Ko S. Y.. (2013b). New paradigm for tumor theranostic methodology using bacteria-based microrobot. Sci. Rep. 3:3394. 10.1038/srep03394 24292152 PMC3844944

[B89] Patra D. Sengupta S. Duan W. Zhang H. Pavlick R. Sen A. (2013). Intelligent, self-powered, drug delivery systems. Nanoscale 5, 1273–1283. 10.1039/C2NR32600K 23166050

[B90] Pilizota T. Brown M. T. Leake M. C. Branch R. W. Berry R. M. Armitage J. P. (2009). A molecular brake, not a clutch, stops the *Rhodobacter sphaeroides* flagellar motor. Proc. Natl. Acad. Sci. U.S.A. 106, 11582–11587. 10.1073/pnas.0813164106 19571004 PMC2710667

[B91] Purcell E. M. (1977). Life at low Reynolds number. Am. J. Phys. 45, 3–11. 10.1119/1.10903

[B92] Qiu T. Lee T. C. Mark A. G. Morozov K. I. Münster R. Mierka O.. (2014). Swimming by reciprocal motion at low Reynolds number. Nat. Commun. 5:5119. 10.1038/ncomms6119 25369018 PMC4241991

[B93] Razin S. Yogev D. Naot Y. (1998). Molecular biology and pathogenicity of mycoplasmas. Microbiol. Mol. Biol. Rev. 62, 1094–1156. 9841667 10.1128/mmbr.62.4.1094-1156.1998 PMC98941

[B94] Ren W. X. Han J. Uhm S. Jang Y. J. Kang C. Kim J.-H.. (2015). Recent development of biotin conjugation in biological imaging, sensing, and target delivery. Chem. Commun. 51, 10403–10418. 10.1039/C5CC03075G 26021457

[B95] Rosser G. Baker R. E. Armitage J. P. Fletcher A. G. (2014). Modelling and analysis of bacterial tracks suggest an active reorientation mechanism in *Rhodobacter sphaeroides*. J. R. Soc. Interface 11:20140320. 10.1098/rsif.2014.0320 24872500 PMC4208361

[B96] Rozhok S. Shen C. K. Littler P. L. H. Fan Z. Liu C. Mirkin C. A.. (2005). Methods for fabricating microarrays of motile bacteria. Small 1, 445–452. 10.1002/smll.200400072 17193470

[B97] Sahari A. Headen D. Behkam B. (2012). Effect of body shape on the motile behavior of bacteria-powered swimming microrobots (BacteriaBots). Biomed. Microdevices 14, 999–1007. 10.1007/s10544-012-9712-1 23053449

[B98] Schliwa M. Woehlke G. (2003). Molecular motors. Nature 422, 759–765. 10.1038/nature01601 12700770

[B99] Schwarz L. Medina-Sánchez M. Schmidt O. G. (2017). Hybrid biomicromotors. Appl. Phys. Rev. 4:031301 10.1063/1.4993441

[B100] Silhavy T. J. Kahne D. Walker S. (2010). The bacterial cell envelope. Cold Spring Harb. Perspect. Biol. 2, 1–16. 10.1101/cshperspect.a000414 20452953 PMC2857177

[B101] Singh A. V. Hosseinidoust Z. Park B. W. Yasa O. Sitti M. (2017). Microemulsion-based soft bacteria-driven microswimmers for active cargo delivery. ACS Nano 11, 9759–9769. 10.1021/acsnano.7b02082 28858477

[B102] Singh A. V. Sitti M. (2016). Bacteria-Driven Particles: patterned and specific attachment of bacteria on biohybrid bacteria-driven microswimmers. Adv. Healthc. Mater. 5:2306 10.1002/adhm.201670097 27240122

[B103] Sockett H. Yamaguchi S. Kihara M. Irikura V. M. Macnab R. M. (1992). Molecular analysis of the flagellar switch protein FliM of *Salmonella typhimurium*. J. Bacteriol. 174, 793–806. 10.1128/jb.174.3.793-806.1992 1732214 PMC206156

[B104] Sokolov A. Apodaca M. M. Grzybowski B. A. Aranson I. S. (2009). Swimming bacteria power microscopic gears. Proc. Natl. Acad. Sci. U.S.A. 107, 969–974. 10.1073/pnas.0913015107 20080560 PMC2824308

[B105] Son K. Guasto J. S. Stocker R. (2013). Bacteria can exploit a flagellar buckling instability to change direction. Nat. Phys. 9, 494–498. 10.1038/nphys2676

[B106] Sowa Y. Berry R. M. (2008). Bacterial flagellar motor. Q. Rev. Biophys. 41, 103–132. 10.1017/S0033583508004691 18812014

[B107] Sowa Y. Hotta H. Homma M. Ishijima A. (2003). Torque–speed relationship of the Na+-driven flagellar motor of *Vibrio alginolyticus*. J. Mol. Biol. 327, 1043–1051. 10.1016/S0022-2836(03)00176-1 12662929

[B108] Stanton M. M. Park B. W. Miguel-López A. Ma X. Sitti M. Sánchez S. (2017a). Biohybrid microtube swimmers driven by single captured bacteria. Small 13, 1–10. 10.1002/smll.201603679 28299891

[B109] Stanton M. M. Park B. W. Vilela D. Bente K. Faivre D. Sitti M.. (2017b). Magnetotactic bacteria powered biohybrids target *E. coli* biofilms. ACS Nano 11, 9968–9978. 10.1021/acsnano.7b04128 28933815

[B110] Stanton M. M. Simmchen J. Ma X. Miguel-López A. Sánchez S. (2016). Biohybrid janus motors driven by *Escherichia coli*. Adv. Mater. Interfaces 3:1500505 10.1002/admi.201500505

[B111] Steager E. Kim C. B. Patel J. Bith S. Naik C. Reber L. (2007). Control of microfabricated structures powered by flagellated bacteria using phototaxis. Appl. Phys. Lett. 90:263901 10.1063/1.2752721

[B112] Steager E. B. Agung Julius A. Kim M. Kumar V. Pappas G. J. Sakar M. S. (2011). Modeling, control and experimental characterization of microbiorobots. Int. J. Rob. Res. 30, 647–658. 10.1177/0278364910394227

[B113] Suh S. Traore M. A. Behkam B. (2016). Bacterial chemotaxis-enabled autonomous sorting of nanoparticles of comparable sizes. Lab Chip 16, 1254–1260. 10.1039/C6LC00059B 26940033

[B114] Swiecicki J.-M. Sliusarenko O. Weibel D. B. (2013). From swimming to swarming: *Escherichia coli* cell motility in two-dimensions. Integr. Biol. 5, 1490–1494. 10.1039/c3ib40130h 24145500 PMC4222179

[B115] Taherkhani S. Mohammadi M. Daoud J. Martel S. Tabrizian M. (2014). Covalent binding of nanoliposomes to the surface of magnetotactic bacteria for the synthesis of self-propelled therapeutic agents. ACS Nano 8, 5049–5060. 10.1021/nn5011304 24684397

[B116] Tao L. Hu W. Liu Y. Huang G. Sumer B. D. Gao J. (2011). Shape-specific polymeric nanomedicine: emerging opportunities and challenges. Exp. Biol. Med. 236, 20–29. 10.1258/ebm.2010.010243 21239732

[B117] Traore M. A. Damico C. M. Behkam B. (2014). Biomanufacturing and self-propulsion dynamics of nanoscale bacteria-enabled autonomous delivery systems. Appl. Phys. Lett. 105:173702 10.1063/1.4900641

[B118] Traoré M. A. Sahari A. Behkam B. (2011). Computational and experimental study of chemotaxis of an ensemble of bacteria attached to a microbead. Phys. Rev. E Stat. Nonlinear Soft Matter Phys. 84:061908. 10.1103/PhysRevE.84.061908 22304117

[B119] Trivedi R. R. Maeda R. Abbott N. L. Spagnolie S. E. Weibel D. B. (2015). Bacterial transport of colloids in liquid crystalline environments. Soft Matter 11, 8404–8408. 10.1039/C5SM02041G 26382153 PMC8968338

[B120] Turner L. Ryu W. S. Berg H. C. (2000). Real-time imaging of fluorescent flagellar filaments. J. Bacteriol. 182, 2793–2801. 10.1128/JB.182.10.2793-2801.2000 10781548 PMC101988

[B121] Turner L. Zhang R. Darnton N. C. Berg H. C. (2010). Visualization of flagella during bacterial swarming. J. Bacteriol. 192, 3259–3267. 10.1128/JB.00083-10 20363932 PMC2897679

[B122] Tuson H. H. Weibel D. B. (2013). Bacteria–surface interactions. Soft Matter 9, 4368–4380. 10.1039/c3sm27705d 23930134 PMC3733390

[B123] Vale R. D. Milligan R. A. (2000). The way things move: looking under the hood of molecular motor proteins. Science 288, 88–95. 10.1126/science.288.5463.88 10753125

[B124] Van Houdt R. Michiels C. W. (2005). Role of bacterial cell surface structures in *Escherichia coli* biofilm formation. Res. Microbiol. 156, 626–633. 10.1016/j.resmic.2005.02.005 15950122

[B125] Vizsnyiczai G. Frangipane G. Maggi C. Saglimbeni F. Bianchi S. Di Leonardo R. (2017). Light controlled 3D micromotors powered by bacteria. Nat. Commun. 8, 1–7. 10.1038/ncomms15974 28656975 PMC5493761

[B126] Vogel P. D. (2005). Nature's design of nanomotors. Eur. J. Pharm. Biopharm. 60, 267–277. 10.1016/j.ejpb.2004.10.007 15939237

[B127] Vollmer W. (2008). Structural variation in the glycan strands of bacterial peptidoglycan. FEMS Microbiol. Rev. 32, 287–306. 10.1111/j.1574-6976.2007.00088.x 18070068

[B128] Vollmer W. Blanot D. De Pedro M. A. (2008). Peptidoglycan structure and architecture. FEMS Microbiol. Rev. 32, 149–167. 10.1111/j.1574-6976.2007.00094.x 18194336

[B129] Wang H. Pumera M. (2015). Fabrication of micro/nanoscale motors. Chem. Rev. 115, 8704–8735. 10.1021/acs.chemrev.5b00047 26234432

[B130] Wang H. Pumera M. (2018). Micro/Nanomachines and Living Biosystems: from simple interactions to microcyborgs. Adv. Funct. Mater. 28:1705421 10.1002/adfm.201705421

[B131] Welch M. Oosawa K. Aizawa S. Eisenbach M. (1993). Phosphorylation-dependent binding of a signal molecule to the flagellar switch of bacteria. Proc. Natl. Acad. Sci. U.S.A. 90, 8787–8791. 10.1073/pnas.90.19.8787 8415608 PMC47445

[B132] Wilchek M. Bayer E. A. Livnah O. (2006). Essentials of biorecognition: the (strept)avidin-biotin system as a model for protein-protein and protein-ligand interaction. Immunol. Lett. 103, 27–32. 10.1016/j.imlet.2005.10.022 16325268

[B133] Williams B. J. Anand S. V. Rajagopalan J. Saif M. T. (2014). A self-propelled biohybrid swimmer at low Reynolds number. Nat. Commun. 5, 1–8. 10.1038/ncomms4081 24435099

[B134] Wolf D. Mascher T. (2016). The applied side of antimicrobial peptide-inducible promoters from Firmicutes bacteria: expression systems and whole-cell biosensors. Appl. Microbiol. Biotechnol. 100, 4817–4829. 10.1007/s00253-016-7519-3 27102123

[B135] Wong D. Beattie E. E. Steager E. B. Kumar V. (2014). Effect of geometry and bacterial collisions on the motion of micro bio robots. Appl. Phys. Lett. 103:153707 10.1063/1.4824840

[B136] Wu Z. Li T. Li J. Gao W. Xu T. Christianson C.. (2014). Turning erythrocytes into functional micromotors. ACS Nano 8, 12041–12048. 10.1021/nn506200x 25415461 PMC4386663

[B137] Xie L. Altindal T. Chattopadhyay S. Wu X.-L. (2011). Bacterial flagellum as a propeller and as a rudder for efficient chemotaxis. Proc. Natl. Acad. Sci.U.S.A. 108, 2246–2251. 10.1073/pnas.1011953108 21205908 PMC3038696

[B138] Young K. D. (2006). The selective value of bacterial shape. Microbiol. Mol. Biol. Rev. 70, 660–703. 10.1128/MMBR.00001-06 16959965 PMC1594593

[B139] Zhang Z. Li Z. Yu W. Li K. Xie Z. Shi Z. (2013). Propulsion of liposomes using bacterial motors. Nanotechnology 24:185103. 10.1088/0957-4484/24/18/185103 23579252

[B140] Zhuang J. Wright Carlsen R. Sitti M. (2015). pH-taxis of biohybrid microsystems. Sci. Rep. 5:11403. 10.1038/srep11403 26073316 PMC4466791

